# Rectal cancer segmentation via HHF-SAM: a hierarchical hypercolumn-guided fusion segment anything model

**DOI:** 10.3389/frai.2025.1696984

**Published:** 2026-01-12

**Authors:** Ye Wang, Ying Yang, Xiaohong Wu, Zhoushan Feng, Congcong Wang

**Affiliations:** 1 Department of Pathology, The First Hospital of China Medical University, Shenyang, China; 2 Department of Nephrology, The Fourth People's Hospital of Shenyang, Shenyang, China; 3 Department of Neonatology, Guangzhou Key Laboratory of Neonatal Intestinal Diseases, The Third Affiliated Hospital of Guangzhou Medical University, Guangzhou, China

**Keywords:** deep learning, medical image analysis, multi-scale, rectal cancer segmentation, segment anything model

## Abstract

**Introduction:**

Rectal cancer is a globally prevalent cancer, and accurate segmentation of rectal lesions in abdominal CT images is critical for clinical diagnosis and treatment planning. Existing methods struggle with imprecise boundary delineation due to low tissue contrast, image noise, and varied lesion sizes, prompting the development of a specialized segmentation framework.

**Methods:**

We developed the Hierarchical Hypercolumn-guided Fusion Segment Anything Model (HHF-SAM) with three core components: 1) A Med-Adapter SAM Encoder integrating LoRA and Adapter modules to adapt SAM's natural image understanding capability to medical-specific features; 2) A Multi-scale Hypercolumn Processing Module to capture comprehensive features for lesions of varying sizes and shapes; 3) A Progressive Hierarchical Fusion Decoder with Hierarchical Fusion Module to aggregate multi-scale features and resolve boundary blurring. The model was evaluated on two public abdominal CT datasets (CARE and WORD) using mean Dice coefficient (mDice) and mean Intersection over Union (mIoU) as metrics.

**Results:**

On the CARE dataset, HHF-SAM achieved a mean mDice of 74.05% and mean mIoU of 58.96%, outperforming state-of-the-art methods (U-SAM: 69.28% mDice, 53.11% mIoU; SAM: 65.98% mDice, 49.44% mIoU). For tumor segmentation specifically, it reached 76.42% mDice and 62.03% mIoU. On the WORD dataset, it achieved an average mDice of 85.84% across all organs, with 83.24% mDice for rectal segmentation (surpassing U-SAM's 80.66% and SAM's 72.77%).

**Discussion:**

This study presents an SAM-based framework optimized for the unique characteristics of abdominal CT images, effectively overcoming the limitations of general segmentation models in medical image processing. The proposed HHF-SAM provides a reliable tool for clinical auxiliary diagnosis, reducing inter-reader variability and improving efficiency in lesion delineation.

## 1 Introduction

Rectal cancer refers to a malignant tumor that occurs in the lining of the rectum and is a type of colorectal cancer. Rectal cancer is one of the more common cancers worldwide, especially in developed countries and regions, where its incidence is relatively high. Early detection of rectal cancer, particularly in its early stages, can significantly improve patient survival rates. Traditional colorectal cancer screening involves inserting a colonoscope into the rectum to examine the inner lining of the colon. However, this method is relatively complex, requiring bowel preparation and posing certain risks and discomfort to the patient. CT colonography is a non-invasive imaging method that uses computed tomography to generate three-dimensional images of the colon, helping doctors detect polyps or cancerous tissue.

However, when faced with a large volume of CT colonography images, the limited availability of radiologists can lead to inaccurate diagnoses, which may have significant consequences for patients. A false-negative diagnosis could delay the optimal treatment window, making treatment more difficult. This is especially critical in cases of malignant diseases like cancer, where time is crucial, and delayed treatment may allow the disease to reach an irreversible stage, endangering the patient's life. Conversely, a false-positive diagnosis may result in patients undergoing unwarranted treatments, which not only offer no therapeutic benefit but also pose the risk of complications and adverse effects.

Deep learning (DL) has drawn increasing attention across various domains, particularly in image recognition and segmentation. When applied to medical CT imaging, deep learning techniques not only enable the precise identification of pathological regions but also facilitate the differentiation between benign and malignant tumors. This capability is crucial for assisting clinicians in making more informed decisions, especially in complex or ambiguous cases, thereby enhancing the overall diagnostic accuracy. Various architectures have been explored for medical image segmentation, each with inherent limitations. CNN-based methods, such as U-Net (Ronneberger, 2015), are adept at automatically extracting features from medical images and excel at capturing fine-grained details through successive layers of convolution and pooling operations. However, due to the local receptive-field characteristics of convolutional kernels, CNNs struggle to summarize global information and manage long-range dependencies. Transformer-based approaches (He et al., 2023; Petit and Thome, 2021) address this limitation by leveraging powerful global modeling capabilities and flexible architectures, achieving robust segmentation results in medical applications. Despite these advantages, Transformers typically require large, annotated datasets and face computational complexity challenges, which limits their effectiveness on tasks such as rectal cancer segmentation, where manually annotated data is scarce.

Recently, the Segment Anything Model (SAM) has gained considerable attention due to its exceptional zero-shot segmentation performance. By leveraging training on over 1.1 billion masks across 11 million natural images, SAM demonstrates proficiency in performing general-purpose image segmentation tasks. In medical image segmentation, there is a growing interest in harnessing SAM to achieve more refined segmentation outcomes. However, several studies (Wald et al., 2023; Shi et al., 2023; Mattjie et al., 2023) have revealed that SAM's zero-shot performance in medical image segmentation remains suboptimal, primarily due to the significant structural differences between natural and medical images. The discrepancy between the training and application domains has led to limited accuracy, with performance being highly sensitive to factors such as dimensions, modality, size, and contrast. While some research (Wu et al., 2023; Chen et al., 2023) has attempted to fine-tune SAM to varying degrees, these approaches entail substantial training costs and pose risks of instability, feature degradation, and catastrophic forgetting.

Considering the aforementioned challenge, we propose an end-to-end learning framework based on SAM, called HHF-SAM, for rectal lesion segmentation. Specifically, we leverage SAM's strong capability in understanding natural images to extract image features. To bridge the gap between natural and medical image domains, we freeze the pre-trained parameters of the SAM encoder and introduce adapter modules to enhance the model's adaptability to medical domain information. Additionally, we incorporate the Multi-scale Hypercolumn Processing Module to improve the model's robustness. This module enables the model to extract multi-scale features from the SAM encoder, which is effective at handling lesions of varying sizes and shapes. Due to the intricate textures in colonoscopy images and the small density differences between soft tissues, distinguishing between them is challenging. The simplistic decoder design in the original SAM struggles to accurately segment lesions. To address this, we propose a Progressive Hierarchical Fusion Decoder that aggregates multi-scale features and provides a more complete representation of the structure in medical images. Extensive experiments on two large abdominal CT image datasets demonstrate that our HHF-SAM framework consistently outperforms other typical segmentation methods.

In summary, our contributions are as follows:

- We propose a novel SAM-based learning framework for rectal cancer segmentation and enhance SAM's adaptability to medical domain information by designing adapter modules.
- We designed a multi-scale hypercolumn processing module that can extract and fuse multi-scale features from the SAM encoder, effectively handling lesion areas of varying shapes and sizes.
- We propose a progressive hierarchical fusion decoder that generates highly accurate, detailed segmentation masks for rectal cancer regions.
- Experimental results demonstrate that, on two large public abdominal CT datasets, our proposed framework outperforms all existing methods in terms of performance.

## 2 Related research

### 2.1 Rectal cancer segmentation

Early methods (Benson et al., 2012; Gambacorta et al., 2013; Kim et al., 2021; Petrillo et al., 2015; Silberhumer et al., 2015) for rectal cancer segmentation from CT images typically relied on global or local thresholding techniques, setting specific gray-level thresholds to distinguish foreground from background. These methods performed well when processing high-contrast, well-structured images, effectively extracting regions of interest. However, when applied to complex medical images, particularly those with noise, overlapping gray values, or blurred tumor boundaries, their performance significantly deteriorated, resulting in inaccurate segmentation. With the rapid advancement of big data and computational power, deep learning techniques have gradually emerged as a powerful tool. By automatically learning complex features from images, deep learning not only significantly improves segmentation accuracy but also demonstrates excellent adaptability, enabling it to handle diverse medical imaging modalities. Today, deep learning methods have become the mainstream approach in CT image segmentation for rectal cancer.

### 2.2 CNN-based medical image segmentation

CNNs have become a dominant approach in medical image segmentation due to their ability to automatically learn hierarchical features from images. Meng et al. (2025) proposed a boundary-constrained mask segmentation network based on CNNs, which effectively reduces the impact of low contrast on the accuracy of medical image segmentation. Sha et al. (2023) proposed a segmentation framework for rectal cancer radiotherapy that uses a registration model to remove noise, thereby enhancing segmentation performance. Zhang et al. (2024) used a traditional UNet to extract global features and incorporated a ResNeSt module to obtain more robust segmentation features. Cai et al. (2024) proposed a segmentation model based on multi-level image features that can more comprehensively capture tumor characteristics, including both fine-grained details and global context. However, due to the local receptive field characteristics of convolutional kernels, CNNs struggle to directly capture a global receptive field. This limitation leads to certain deficiencies in handling long-range dependencies or global contextual information. While the receptive field can be expanded by increasing the network's depth and number of layers, this approach is generally inefficient and may lead to issues such as vanishing gradients or information loss.

### 2.3 Transformer-based medical image segmentation

Transformer architecture has demonstrated remarkable success in medical image segmentation by capturing long-range dependencies and global context. Sang et al. (2024) proposed a network architecture, FCTformer, that integrates convolutional operations with Transformer modules to achieve precise segmentation of rectal tumors in 3D MRI. Meng et al. (2025) designed a boundary-constrained multi-task learning network that can automatically localize and segment both rectal cancer and the rectal wall. Tan et al. (2023) introduced a Transformer-based multiple-instance learning framework that combines global and local features to achieve high-accuracy lymph node detection. Liu et al. (2023) combined CNNs with Transformer to develop a parallel hybrid network architecture, which efficiently segments skin melanomas and has also achieved remarkable results in the segmentation of colon polyps with ambiguous boundaries. Sun et al. (2024) proposed the DA-TransUNet, which integrates the Transformer and dual attention blocks into the traditional U-shaped architecture. It optimizes the intermittent channels of dual attention and applies it to each skip connection to effectively filter out irrelevant information. Lan et al. (2024) proposed BRAUNet++, which reconstructs skip connections using bi-level routing attention and channel-spatial attention, and employs a hierarchical U-shaped encoder-decoder structure to learn global semantic information while reducing computational complexity and enhancing the interaction of global dimensions across multi-scale features. However, the precise segmentation results produced by Transformer typically rely on large, annotated datasets. In the task of rectal cancer segmentation, the limited size of manually annotated datasets often constrains the effectiveness and potential of the Transformer.

### 2.4 SAM-based medical image segmentation

To bridge the gap between natural and medical images, several studies have explored adaptive strategies to improve SAM's performance in medical image segmentation tasks. He et al. (2023) evaluated SAM's zero-shot capabilities across 12 public medical image segmentation datasets, revealing that its performance is highly sensitive to factors such as dimensions, modality, size, and contrast. Yan et al. (2024) proposed the AFTer-SAM architecture, which optimizes SAM through low-rank adaptation. It also leverages axial fusion transformers to seamlessly integrate intra- and inter-slice contextual information, significantly enhancing segmentation performance on medical images. Xie et al. (2024) introduced a few-shot fine-tuning strategy that reconstructs the mask decoder within SAM. It uses derived few-shot embeddings as prompts to segment objects captured in the query image embeddings, thereby improving segmentation accuracy. Cheng et al. (2024) proposed the H-SAM architecture, an adaptive SAM algorithm based on a two-stage hierarchical decoding process, enabling efficient fine-tuning for medical images. Paranjape et al. (2024) introduced an adaptive strategy for S-SAM that enables the generation of precise segmentation masks for medical images. Although these SAM-based approaches have achieved commendable segmentation accuracy, they still suffer from performance degradation and limited generalization when handling low-contrast samples, indistinct boundaries, complex shapes, or small sizes.

## 3 Math

**Figure 1:**
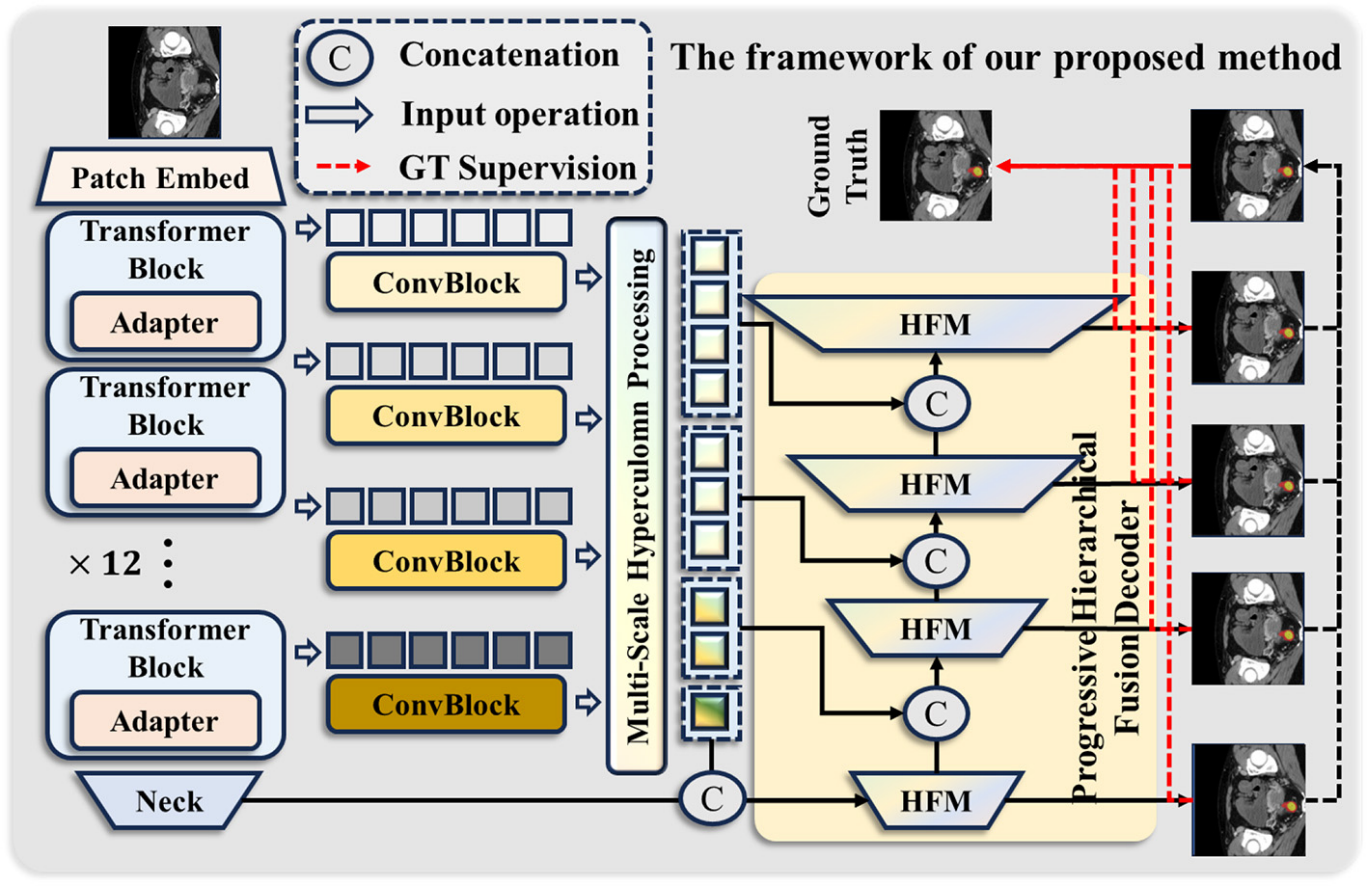
Overview of the proposed Hierarchical Hypercolumn-guided Fusion Segment Anything Model (HHF-SAM) for rectal cancer segmentation. Best viewed by zooming in.

As illustrated in Figure 1, the overall framework of our method comprises three key modules: the Med-Adapter SAM Encoder (MSE), the Multi-scale Hypercolumn Processing Module (MHPM), and the Progressive Hierarchical Fusion Decoder (PHFD). Each module will be explained in detail in the subsections that follow.

### 3.1 Med-adapter SAM encoder

**Figure 2:**
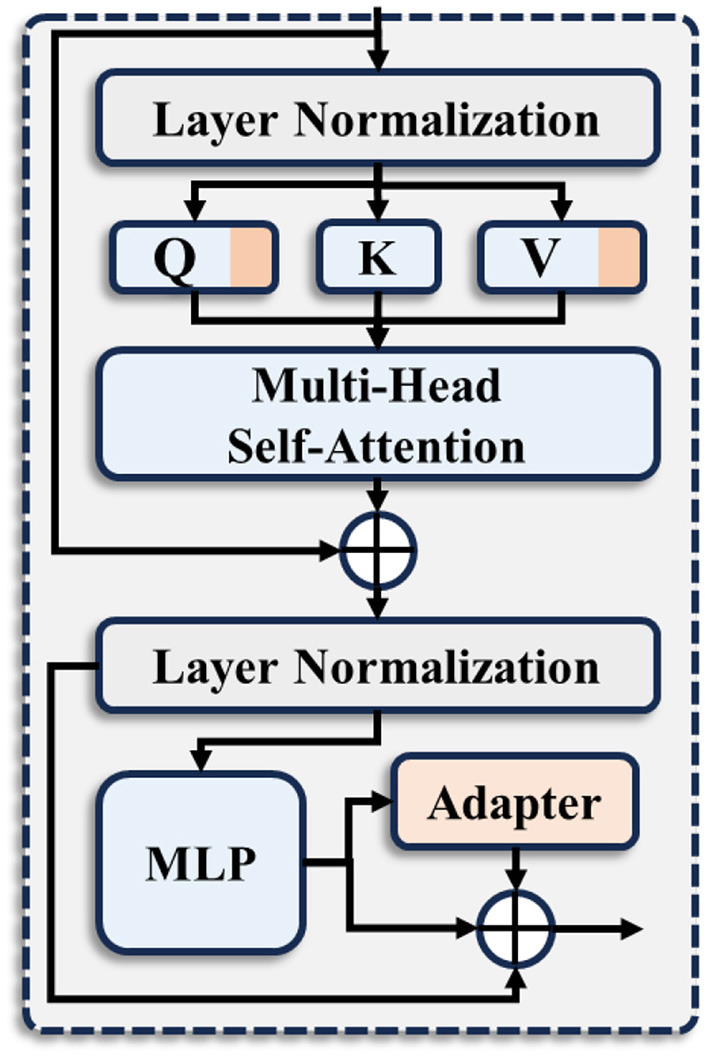
The enhanced transformer block in our proposed Med-Adapter SAM Encoder (MSE).

SAM has been pre-trained on a large-scale dataset, learning rich feature representations. Using it as a backbone network allows us to fully leverage these pre-trained features, improving the model's convergence speed and performance on downstream tasks. However, due to the significant differences between natural and medical images, directly applying the pre-trained SAM to medical image segmentation tasks is not optimal. Therefore, we propose a SAM encoder specifically designed for medical image segmentation. As shown in Figure 2, we retain the core components of the original SAM encoder and keep its parameters fixed during training. Additionally, to bridge the gap between natural and medical images, we incorporate LoRA (Hu et al., 2021) and Adapter (Houlsby et al., 2019) within the Transformer. More specifically, let ${\text{X}}_{i}\in {\mathbb{R}}^{N\times D}$ be the input of the *i*-th Transformer block. where *N* is the number of tokens and *D* denotes the embedding dimension. The output of the MHSA layer can be expressed as follows:


$$\begin{array}{l} {\text{Q}}_{i}=W_{q}({\text{X}}_{i})+W_{q}^{up}(W_{q}^{down}({\text{X}}_{i}), \end{array}$$ (1)

$$\begin{array}{l} {\text{K}}_{i}=W_{k}\left({\text{X}}_{i}\right), \end{array}$$ (2)

$$\begin{array}{l} {\text{V}}_{i}=W_{v}({\text{X}}_{i})+W_{v}^{up}(W_{v}^{down}({\text{X}}_{i}), \end{array}$$ (3)

$$\begin{array}{l} {\bar{\text{X}}}_{i}=MHSA\left({\text{Q}}_{i},{\text{K}}_{i},{\text{V}}_{i}\right)+{\text{X}}_{i}, \end{array}$$ (4)

where *W*_*q*_, *W*_*k*_, and *W*_*v*_ are the weights of three linear projection layers used to generate the original *Query*, *Key*, and *Value* matrices, respectively. $W_{q,v}^{down}$and $W_{q,v}^{up}$ are the weights of two linear projections that constitute LoRA. The parameters of LoRA are learnable during training. Additionally, the output of the i-th Transformer layer can be expressed as follows:


$$\begin{array}{l} {\hat{\text{X}}}_{i}=MLP\left(LN\left({\bar{\text{X}}}_{i}\right)\right), \end{array}$$ (5)

$$\begin{array}{l} {\text{Y}}_{i}=W_{adpt}^{up}\left(\sigma \left(W_{adpt}^{down}\left({\hat{\text{X}}}_{i}\right)\right)\right)+{\bar{\text{X}}}_{i}, \end{array}$$ (6)

where *LN* and *MLP* stand for the Layer Normalization (LN) and Multilayer Perceptron (MLP), respectively. σ represents the Rectified Linear Unit (ReLU). $W_{adpt}^{down}\in {\mathbb{R}}^{P\times D}$ and $W_{adpt}^{up}\in R^{P\times D}$ are the weights of two linear projections. The adapter not only includes these two linear projections but also incorporates a ReLU activation function to enhance its expressive capability. By freezing the original parameters of the SAM encoder while unfreezing the parameters of LoRA and Adapter, we can fully leverage the pre-trained features of SAM on large-scale datasets. This approach helps mitigate the significant differences between natural and medical images, enabling the extraction of more robust feature information.

### 3.2 Multi-scale hypercolumn processing module

Colonoscopy images are characterized by low contrast and complex structures, with lesion areas that are uncertain in scope and variable in size. To address this challenge, we propose an MHPM that extracts features from both spatial and channel dimensions, enabling a more efficient capture of key information in these images. Specifically, given an input image X ∈ ℝ^*H* × *W* × 3^, we pass it through the SAM encoder, extracting features from the 3rd, 6th, 9th, and 12th layers of the Transformer as Y_*i*_(*i* = 3, 6, 9, 12). In general, shallow layers capture fine-grained details, while deeper layers capture more semantic information. We then reshape these features into spatial feature maps and input them into the MHPM.

**Figure 3:**
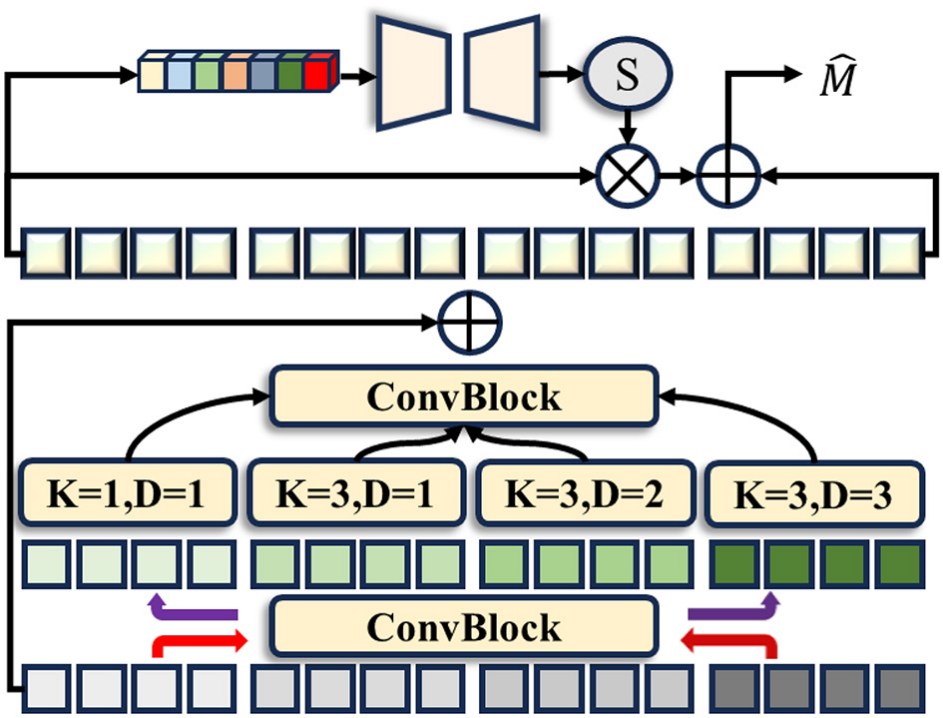
The structure of our proposed multi-scale Hypercolumn Processing Module (MHPM).

The architecture of the MHPM is depicted in Figure 3. Initially, a convolutional layer is employed to reduce the number of channels to one-quarter of Y_*i*_. Subsequently, four dilated convolutional layers are used to extract multi-scale features, progressively expanding the receptive fields. The features from these four branches are concatenated along the channel dimension, followed by a convolutional layer to aggregate them. Finally, a residual connection is introduced to generate the HEM module's final output, ensuring efficient feature fusion.


$$\begin{array}{l} \bar{\text{H}}={\text{Conv}}_{1\times 1}\left({\text{Y}}_{\text{i}}\right), \end{array}$$ (7)

$$\begin{array}{l} {\text{H}}_{1}={\text{Conv}}_{1\times 1,\text{d}=1}\left(\bar{\text{H}}\right),{\text{H}}_{2}={\text{Conv}}_{3\times 3,\text{d}=1}\left(\bar{\text{H}}\right), \end{array}$$

$$\begin{array}{l} {\text{H}}_{3}={\text{Conv}}_{3\times 3,\text{d}=2}\left(\bar{\text{H}}\right),{\text{H}}_{4}={\text{Conv}}_{3\times 3,\text{d}=3}\left(\bar{\text{H}}\right), \end{array}$$ (8)

$$\begin{array}{l} \text{H}={\text{Y}}_{i}+{\text{Conv}}_{1\times 1}\left(\left[{\text{H}}_{1};{\text{H}}_{2};{\text{H}}_{3};{\text{H}}_{4}\right]\right), \end{array}$$ (9)

where *d* represents the dilation rate. The dilation rates of 1, 1, 2, and 3 are specifically chosen to create a gradual expansion of receptive fields. The first two branches (*d* = 1) capture fine-grained local details, while the latter branches (*d* = 2, 3) progressively enlarge the receptive field to capture broader contextual information. This configuration balances the trade-off between capturing local texture details and global structural patterns, which is particularly important for segmenting rectal lesions with varying sizes and irregular boundaries. We further integrate features along the channel dimension. By dynamically assigning different weights to each channel, the model becomes better able to focus on features relevant to the target task. The hypermap M_*i*_ can be expressed as follows:


$$\begin{array}{l} \hat{\text{M}}=\text{H}\times \delta \left({\text{Conv}}_{1\times 1}\left(\text{GAP}\left(\text{H}\right)\right)\right)+\text{H}, \end{array}$$ (10)

$$\begin{array}{l} {\text{M}}_{i}={\text{Conv}}_{3\times 3}\left(\psi \left(\hat{\text{M}}\right)\right). \end{array}$$ (11)

where *GAP* stands for the Global Average Pooling, δ represents the Sigmoid function, and ψ is a deconvolutional layer. By using convolutional kernels of various sizes and dilation rates, the model can capture features at various scales. Further integration of these multi-receptive field features along the channel dimension enables the model to gain a more comprehensive understanding of the image, thereby improving overall performance.

Regarding the multi-scale hypercolumn fusion process: (1) Spatial alignment: since all features extracted from different Transformer layers share the same spatial resolution of (*H*/16) × (*W*/16), no additional spatial alignment or interpolation is required before concatenation; (2) Channel normalization: Batch Normalization (BN) is applied after each convolutional layer in the MHPM to normalize feature distributions across channels, ensuring stable training and preventing feature scale discrepancies; (3) Feature scale handling: the channel attention mechanism (Equation 8) dynamically assigns weights to different channels based on their global statistics, effectively handling variations in feature scales and distributions from different encoder layers.

### 3.3 Progressive hierarchical fusion decoder

**Figure 4:**
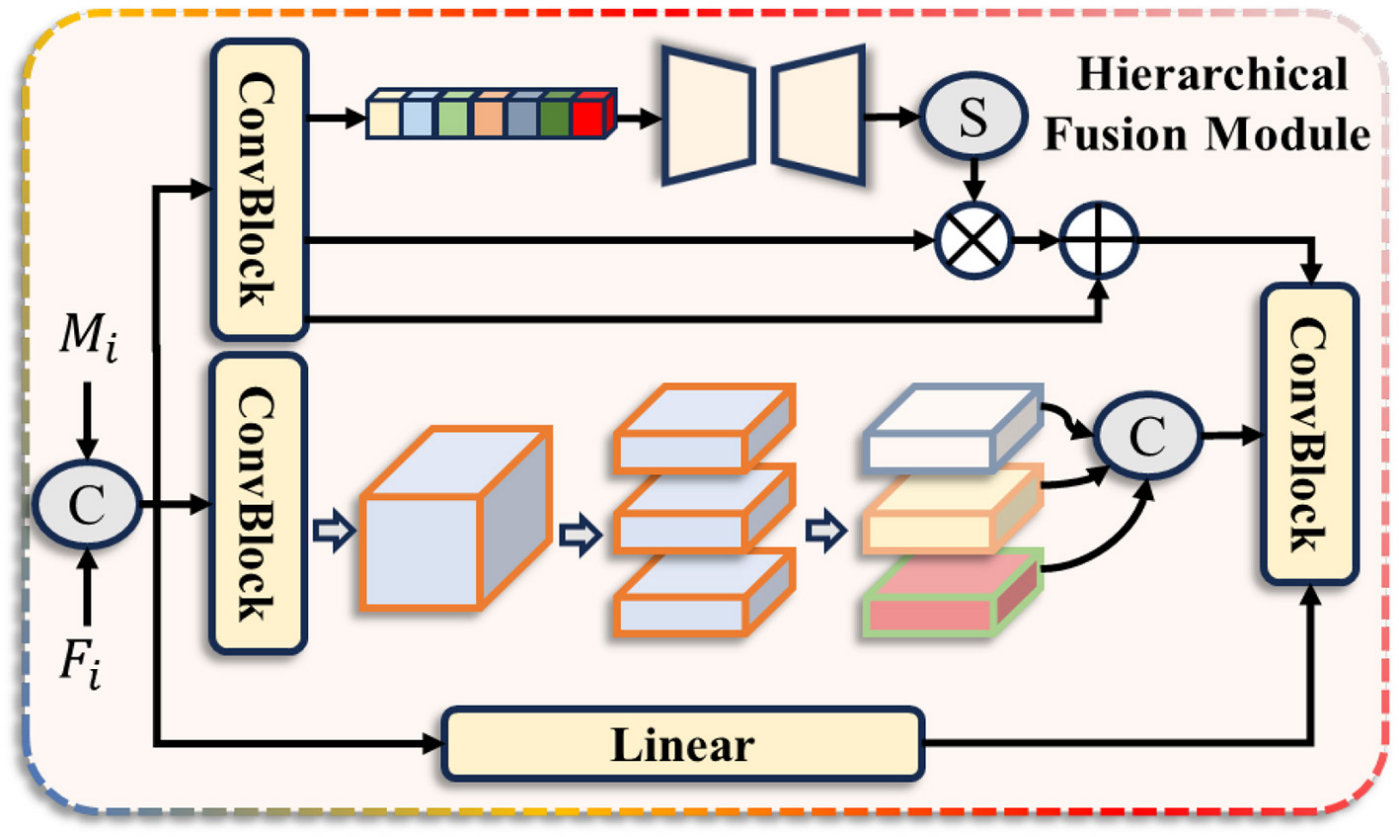
The structure of our Hierarchical Fusion Module (HFM).

Colonoscopy images contain a large amount of intricate, complex textures, particularly subtle density differences among soft tissues, making them difficult to distinguish. The simple decoder design in the original SAM struggles to accurately segment lesion areas. To address this issue, we propose a Progressive Hierarchical Fusion Decoder for efficient segmentation predictions. This decoder adopts a pyramidal structure, progressively integrating features from the SAM encoder and the Hierarchical Fusion Module (HFM) to generate precise segmentation results. As shown in Figure 1, we concatenate the output of the MHPM with the output of the previous-level HFM along the channel dimension and then use the next-level HFM for further feature enhancement. Thus, the output of the i-th stage of the pyramid can be expressed as follows:


$$\begin{array}{l} {\text{F}}_{j+1}=\text{HFM}\left({\text{Conv}}_{1\times 1}\left[{\text{M}}_{\text{i}};{\text{F}}_{\text{i}}\right]\right),\text{j}=1,2,3. \end{array}$$ (12)

where *M*_*i*_ represents the output of the MHPM, and *F*_*i*_ represents the output of the previous-level HFM. HFM refers to the Hierarchical Fusion Module, and its structure is shown in Figure 4. This module enhances the model's feature representation capability by integrating features at three levels: global, regional, and local, thereby capturing richer and more profound semantic information. Specifically, let D_*i*_ be the input of the HFM module at the i-th stage of the pyramid. Then, the output of HFM can be expressed as follows:


$$\begin{array}{l} {\text{F}}_{Global}={\text{D}}_{i}\times \delta \left({\text{Conv}}_{1\times 1}\left(\text{GAP}\left({\text{D}}_{\text{i}}\right)\right)\right)+{\text{D}}_{\text{i}}, \end{array}$$ (13)

$$\begin{array}{l} {\text{F}}_{Region}^{k}={\text{D}}_{i}^{k}\times \delta \left({\text{Conv}}_{1\times 1}\left(\text{GAP}\left({\text{D}}_{\text{i}}^{\text{k}}\right)\right)\right)+{\text{D}}_{\text{i}}^{\text{k}}, \end{array}$$ (14)

$$\begin{array}{l} {\text{F}}_{Region}=\left[{\text{F}}_{Region}^{1};{\text{F}}_{Region}^{2};...;{\text{F}}_{Region}^{k}\right], \end{array}$$ (15)

$$\begin{array}{l} {\text{F}}_{Local}=\text{MLP}\left({\text{D}}_{\text{i}}\right), \end{array}$$ (16)

$$\begin{array}{l} {\text{F}}_{i}={\text{Conv}}_{1\times 1}\left(\left[{\text{F}}_{\text{Global}};{\text{F}}_{\text{Region}};{\text{F}}_{\text{Local}}\right]\right). \end{array}$$ (17)

where ${\text{D}}_{i}^{k}$ represents the division of D_*i*_ into *K* groups along the channel dimension, and MLP represents the linear projection. This grouping method effectively prevents the model from relying too heavily on any single channel, thereby enhancing its generalization. Through the synergistic effect of the pyramidal structure and HFM, our framework can generate highly refined, detailed segmentation masks for lesions of varying shapes and sizes.

Our PHFD differs from existing pyramid-style decoders in several key aspects: (1) Unlike U-Net++, which uses dense nested skip connections, PHFD employs a progressive fusion strategy that explicitly combines multi-scale hypercolumn features with hierarchical decoder outputs; (2) Unlike HRNet, which maintains high-resolution representations throughout, PHFD focuses on efficient feature aggregation through the HFM module that captures global, regional, and local information simultaneously; (3) Compared to FPN, which uses top-down feature propagation with lateral connections, PHFD incorporates channel-wise attention mechanisms within HFM to dynamically weight features based on their relevance to the segmentation task.

### 3.4 Loss function

To fully optimize the proposed framework, we introduced multi-scale supervision signals to the outputs of each layer in the model's decoder. Additionally, we integrated the predictions from the previous layers to obtain the final prediction results using the following formula:


$$\begin{array}{l} F={\text{Conv}}_{1\times 1}\left(\left[{\text{F}}_{1};{\text{F}}_{2};{\text{F}}_{3};F_{4}\right]\right), \end{array}$$ (18)

where *F*_*k*_, (*k* = 1, 2, 3, 4) refers to the prediction result at the i-th stage of the pyramid. To ensure the classification accuracy of each pixel, we employed the cross-entropy loss function for supervision, as shown in the following equation:


$$\begin{array}{r} L=-\sum_{i=1}^{H}\sum_{j=1}^{W}[GT_{i,j}\ln\left(\delta \left(P_{i,j}\right)\right)+\\ \left(1-GT_{i,j}\right)\ln\left(1-\delta \left(P_{i,j}\right)\right)], \end{array}$$ (19)

where *GT* refers to the ground truth. Through the aforementioned supervision, the network parameters were thoroughly optimized. This optimization significantly improved the model's ability to capture fine-grained details across various scales, resulting in superior performance in segmenting rectal cancer lesions.

## 4 Experiments

### 4.1 Datasets and evaluation metrics

To validate the performance of the proposed model, we trained and tested it on two publicly available large-scale abdominal CT datasets. The CARE dataset (Wan et al., 2024) was annotated in detail by more than ten experienced gastrointestinal surgeons, who meticulously outlined the diseased and normal rectal regions layer by layer. This dataset is divided into two subsets: the training set contains 318 samples with a total of 36,563 slice pairs, and the test set contains 81 samples with 6,461 slice pairs. The WORD dataset (Luo et al., 2021) is a large-scale abdominal organ segmentation dataset comprising 150 scans spanning the entire abdominal region, totaling 30,495 slices. Among them, 100 scans are used for training, 20 for validation, and 30 for testing. The evaluation on these two datasets demonstrates the robustness and practical effectiveness of the proposed model.

We used the mean Dice coefficient (mDice) and mean intersection over Union (mIoU) to quantitatively evaluate the model's performance. mDice is a widely used metric in image segmentation tasks. It measures the similarity between predicted segmentation results and ground-truth labels, effectively reflecting overall segmentation performance.


$$\begin{array}{l} Dice\left(A,B\right)=\frac{2|A\cap B|}{\left|A|+|B\right|} \end{array}$$ (20)

$$\begin{array}{l} mDice=\frac{1}{C}\overset{C}{\underset{i=1}{\sum }}Dice_{i}, \end{array}$$ (21)

where *A* is the ground truth binary mask, *B* is the predicted binary mask, |*A* ∩ *B*| represents the common elements between sets A and B, and |*A*| + |*B*| denotes the total number of elements in A and B, respectively. mIoU is a more stringent metric, as it focuses solely on the overlapping regions between the predicted and actual areas, making it particularly well-suited for evaluating segmentation accuracy, especially in multi-class scenarios.


$$\begin{array}{l} IoU=\frac{\left|A\cap B\right|}{\left|A\cup B\right|} \end{array}$$ (22)

$$\begin{array}{l} mIoU=\frac{1}{C}\overset{C}{\underset{i=1}{\sum }}IoU_{i}, \end{array}$$ (23)

where |*A* ∪ *B*| represents the total number of pixels covered by A, B, or both.

### 4.2 Implementation details

We implemented the model using the PyTorch toolkit and conducted experiments on two RTX 3090 GPUs, each equipped with 24 GB of video memory. For the backbone network of the model, we adopted the SAM-B weights pre-trained on natural images and froze their parameters, fine-tuning only the other parts. To enhance data diversity and improve the model's generalization, we applied two common data augmentation methods: random flipping and random rotation. Consistent with previous work (Oktay et al., 2018; Jha et al., 2019; Ibtehaz and Rahman, 2020), all input images were resized to 224 × 224. Due to memory limitations, we set the batch size to 16. We employed the widely recognized AdamW optimizer for parameter updates, with an initial learning rate of 0.001 and a weight decay coefficient of 0.1. To improve convergence, the learning rate was reduced by a factor of 10 every 20 epochs, and the training process spanned 50 epochs. Our code will be made publicly available to enable other researchers to reproduce our results and further optimize the model.

### 4.3 Comparison with state-of-the-arts

In this section, we compare the proposed method with other state-of-the-art methods on two large abdominal CT datasets. Consistent with previous work, we use the Dice coefficient and Intersection over Union (IoU) as evaluation metrics for the CARE dataset, while reporting the Dice coefficient for all organs in the WORD dataset. Tables 1, 2 present the quantitative comparison results for the CARE and WORD datasets, respectively. AttenUnet (Oktay et al., 2018) introduces an attention gate (AG) module, which can implicitly learn to suppress irrelevant regions in the input image while highlighting salient features relevant to the specific task. The AG module is plug-and-play and can significantly enhance model sensitivity and prediction accuracy with minimal computational overhead. ResUNet++ (Jha et al., 2019) presents an improved ResUNet architecture for colonoscopic image segmentation, incorporating residual units, squeeze-and-excitation units, ASPP, and attention units. This architecture has demonstrated outstanding segmentation performance on public datasets. MultiResUNet (Ibtehaz and Rahman, 2020) introduces a lightweight, memory-efficient MultiRes module that significantly improves the model's segmentation performance on complex images. Although the segmentation results may not be perfect in extreme cases, the model demonstrates substantial improvements over the classical U-Net in most situations. MISSFormer (Huang et al., 2022) proposes a U-shaped Transformer encoder that enhances feature discrimination by reintegrating local contextual information and global dependencies. Additionally, a ReMixed Transformer Context Bridge is introduced into the decoder to further improve fine-grained segmentation accuracy. SwinUnet (Cao et al., 2022) uses a hierarchical Swin Transformer as the encoder to extract contextual features via a shifted-window mechanism. It incorporates a symmetric Swin Transformer-based decoder, combined with expanded layers for upsampling operations, to restore the spatial resolution of feature maps. The model excels at medical image segmentation tasks, effectively learning global semantic information and long-range dependencies, yielding superior segmentation performance. TransUNet (Chen et al., 2021) employs a Transformer encoder, combined with a U-Net to preserve local spatial information, and ultimately demonstrates excellent performance in medical applications such as multi-organ segmentation. UCTransNet (Wang et al., 2022) proposes a Transformer-based segmentation model from a channel-wise perspective, incorporating an attention mechanism and integrating recurrent neural networks and channel-wise cross-attention. This approach ultimately achieved excellent results across multiple medical image segmentation datasets. nnU-Net (Isensee et al., 2021) can automatically configure its network architecture, training strategies, and preprocessing steps based on the given dataset, significantly reducing the complexity of deep learning applications. SAM (Kirillov et al., 2023) is a universal, promptable image segmentation model that achieves efficient segmentation of any object by combining large-scale data and advanced model architecture, significantly expanding the application scope and convenience of image segmentation. SAM+LoRA (Zhang, 2023) proposed a LoRA fine-tuning strategy and, together with the prompt encoder and mask decoder, fine-tuned on medical image segmentation datasets. This successfully improved SAM's performance in medical image segmentation tasks. AFTer-SAM (Yan et al., 2024) introduced adapter modules into SAM and leveraged axial fusion transformers to integrate contextual information, thereby improving performance on medical images. U-SAM (Wan et al., 2024) proposed a U-shaped adapter architecture, correcting the inherent structural limitations of SAM when applied to medical image analysis. This architecture significantly improves the efficiency and accuracy of rectal cancer diagnosis in clinical practice.

**Table 1:** Performance comparison on the CARE dataset.

**Methods**	**Normal**		**Tumor**		**Mean**	
	**mDice (**%**)**	**mIoU (**%**)**	**mDice (**%**)**	**mIoU (**%**)**	**mDice (**%**)**	**mIoU (**%**)**
AttenUnet (Oktay et al., 2018)	63.05	46.04	71.39	55.50	67.22	50.77
ResUnet++ (Jha et al., 2019)	58.08	40.93	69.87	53.69	63.97	47.31
MultiResUnet (Ibtehaz and Rahman, 2020)	62.25	45.19	72.11	56.39	67.18	50.79
MissFormer (Huang et al., 2022)	53.63	36.64	68.58	52.19	61.11	44.41
SwinUnet-B (Cao et al., 2022)	63.32	46.32	72.63	57.02	67.97	51.67
SwinUnet-L (Cao et al., 2022)	61.66	44.57	72.58	56.97	67.12	50.77
TransUnet-B (Chen et al., 2021)	60.21	43.08	70.69	54.67	65.45	48.87
TransUnet-L (Chen et al., 2021)	63.75	46.79	72.60	56.98	68.17	51.86
UCTransNet (Wang et al., 2022)	63.53	46.55	70.67	54.64	67.10	50.59
nnUNet (Isensee et al., 2021)	59.73	43.68	72.00	57.62	65.86	50.65
SAM (Kirillov et al., 2023)	60.95	43.83	71.00	55.04	65.98	49.44
SAM+LoRA (Zhang, 2023)	57.57	40.42	70.70	54.68	64.14	47.55
AFTer-SAM (Yan et al., 2024)	62.35	45.12	71.58	55.87	66.97	50.50
U-SAM (Wan et al., 2024)	65.72	48.94	72.84	57.28	69.28	53.11
**HHF-SAM**	**72.14**	**56.24**	**76.42**	**62.03**	**74.05**	**58.96**

The table shows evaluation metrics for different methods. The best results for each metric are highlighted in bold. Our proposed method achieves superior performance compared to existing approaches.

**Table 2:** Performance comparison on the WORD dataset.

**Methods**	**Liver**	**Spleen**	**Kidney(L)**	**Kidney(R)**	**Stomach**	**Gallbladder**	**Esophagus**	**Pancreas**	**Duodenum**	**Colon**	**Intestine**	**Adrenal**	**Rectum**	**Bladder**	**HFL**	**HFR**	**Mean**
FocalUnet (Naderi et al., 2022)	93.21	89.54	88.64	88.68	83.43	61.29	57.83	60.57	45.20	70.72	72.47	48.03	70.08	90.47	84.63	83.77	74.28
R2Unet (Alom et al., 2019)	84.73	90.35	90.56	87.78	80.21	59.56	71.12	72.64	49.74	70.77	73.30	48.26	72.99	88.20	74.17	47.78	72.63
ResUnet++ (Jha et al., 2019)	95.08	93.71	93.92	94.22	89.28	69.28	72.95	75.82	57.15	79.80	80.73	65.59	75.27	93.20	**92.26**	**92.01**	82.52
MultiResUnet (Ibtehaz and Rahman, 2020)	95.19	93.73	93.12	93.33	90.73	69.83	73.11	75.33	60.36	81.32	82.51	64.51	78.35	93.57	85.25	87.94	82.30
MissFormer (Huang et al., 2022)	85.65	94.60	91.00	91.30	90.22	71.62	72.27	76.02	57.85	80.44	80.87	64.02	76.55	93.53	87.26	86.90	81.89
SwinUnet-B (Cao et al., 2022)	94.91	91.73	89.80	89.76	90.43	70.05	72.33	74.01	56.69	79.85	80.47	61.67	78.01	93.27	87.71	87.82	81.16
SwinUnet-L (Cao et al., 2022)	95.19	92.69	89.87	89.94	90.45	72.97	72.66	72.89	58.37	79.67	80.51	59.77	77.55	93.63	87.76	87.69	81.37
TransUnet-B (Chen et al., 2021)	95.46	93.21	91.47	91.63	90.01	70.99	70.61	75.38	55.47	78.73	81.25	64.74	76.66	93.76	87.12	87.56	81.50
TransUnet-L (Chen et al., 2021)	94.93	89.88	90.56	90.47	91.62	95.52	75.17	76.51	60.41	81.78	83.18	67.33	79.63	94.33	88.40	88.07	82.99
UCTransNet (Wang et al., 2022)	95.19	94.18	94.27	94.62	89.04	65.83	68.67	73.30	58.44	79.60	80.59	64.36	75.43	92.23	89.31	89.79	81.55
nnUNet (Isensee et al., 2021)	95.44	93.91	94.55	94.60	89.63	66.56	74.78	78.85	63.57	82.45	85.41	65.85	72.42	92.42	84.76	77.58	82.05
SAM (Kirillov et al., 2023)	94.50	91.67	89.44	88.91	87.77	59.83	61.90	70.15	51.53	71.91	75.83	51.71	72.77	91.91	88.24	88.34	77.28
U-SAM (Wan et al., 2024)	95.47	94.94	95.33	95.46	91.66	76.91	77.91	75.58	65.60	83.38	83.27	69.39	80.66	94.20	88.23	88.31	84.83
**HHF-SAM**	**96.12**	**95.03**	**95.41**	**95.87**	**92.17**	**77.87**	**82.21**	**80.37**	**66.03**	**83.72**	**84.41**	**73.85**	**83.24**	**95.45**	92.21	91.18	**85.84**

Higher values reflect better performance across all evaluation metrics. The best results for each metric are highlighted in bold.

The aforementioned methods have all demonstrated strong performance in rectal cancer segmentation tasks. However, our approach fully leverages the rich semantic information embedded in SAM's pre-trained weights, combined with a multi-scale feature enhancement module and a refined pyramid decoder structure. This enables our model to segment fine-grained lesion areas more accurately. Compared to adapter-based methods like AFTer-SAM, which primarily focus on domain adaptation, our HHF-SAM additionally extracts multi-scale hypercolumn features from different encoder layers, enabling richer spatial-semantic representation. Furthermore, unlike U-SAM, which employs a U-shaped decoder structure, our Progressive Hierarchical Fusion Decoder systematically aggregates features through the HFM module, which simultaneously captures global, regional, and local information, thereby providing more refined boundary delineation for complex lesion shapes. Results on two large abdominal CT datasets show that our model significantly outperforms the previously mentioned techniques.

### 4.4 Qualitative comparisons

**Figure 5:**
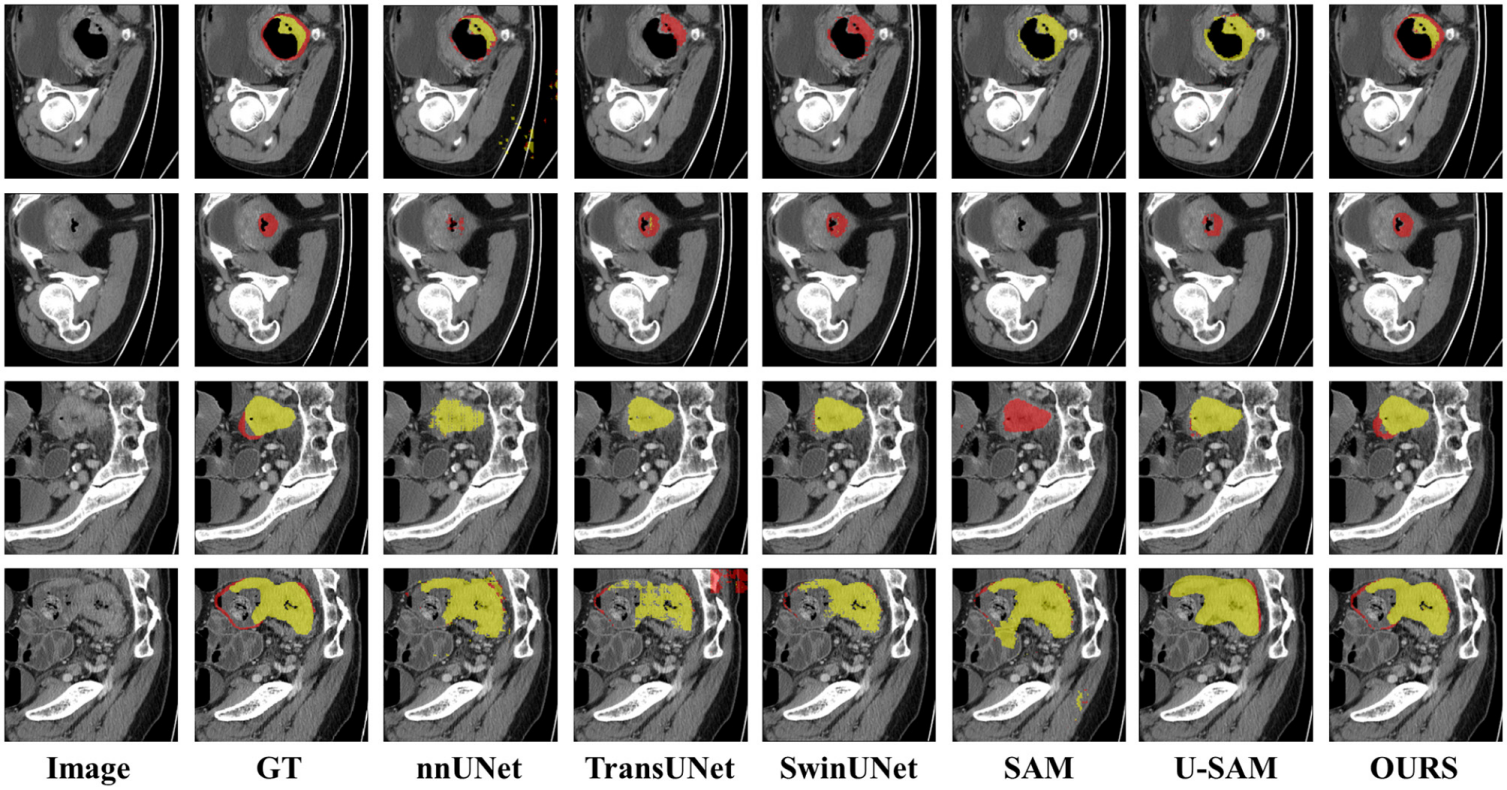
Visual comparisons of different methods on the CARE dataset. The results illustrate the segmentation performance of each method. Best viewed by zooming in.

**Figure 6:**
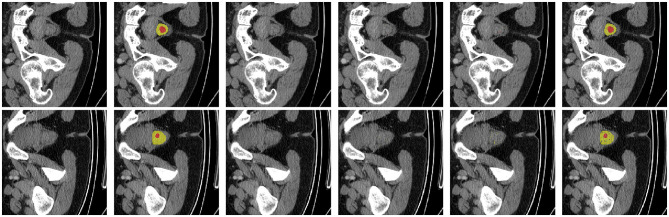
Failure case analysis on challenging samples. **(Left to right)** Original image, ground truth, SAM, AFTer-SAM, U-SAM, and our HHF-SAM. Yellow represents normal rectal tissue, and red indicates tumor regions. Our method achieves better segmentation even in these difficult cases.

To facilitate a more intuitive comparison of segmentation performance across models, we have visualized each model's output features in Figure 5. Specifically, yellow denotes normal rectal tissue, while red denotes rectal cancer tumors; the boxes highlight the regions where the models' predictions differ from the ground-truth annotations. Evidently, compared to other models, our model achieves more refined segmentation while preserving intricate shape information. Regarding the boundaries between tumors and healthy rectal walls, our model's segmentation results are also closer to the ground truth annotations. We acknowledge that in Rows 1 and 2 of Figure 5, none of the compared methods, including ours, achieve complete lesion coverage. This is primarily due to the inherent challenges in these cases: (1) the lesions exhibit extremely low contrast with surrounding tissues, making boundary delineation difficult; (2) the lesion shapes are highly irregular and diffuse, which poses challenges for all segmentation methods. However, our HHF-SAM still demonstrates relatively better performance in capturing the main lesion regions compared to other methods. These challenging cases also highlight the need for future research on handling low-contrast and diffuse lesions in clinical applications. To validate the effectiveness of the proposed architecture, we further visualized the features of each module. As illustrated in Figure 6, the segmentation regions become increasingly detailed as our key modules are progressively applied. This further underscores the significant advantages of our proposed model.

### 4.5 Failure case analysis

To provide a comprehensive evaluation of our method, we present a failure case analysis in Figure 6. These cases represent challenging scenarios where most methods struggle to achieve accurate segmentation. As shown in the figure, the original SAM (Kirillov et al., 2023) tends to produce over-segmented results due to its lack of domain-specific knowledge for medical imaging. AFTer-SAM (Yan et al., 2024) and U-SAM (Wan et al., 2024) show improved localization but still fail to capture the complete tumor regions accurately. In contrast, our HHF-SAM demonstrates superior performance even in these difficult cases, owing to its multi-scale hypercolumn features and the progressive hierarchical fusion mechanism. The red regions in the last column indicate the remaining tumor areas that our method successfully captures, while other methods miss them. These results highlight the robustness of our approach in handling challenging cases with low contrast and irregular lesion boundaries.

### 4.6 Ablation study

**Figure 7:**
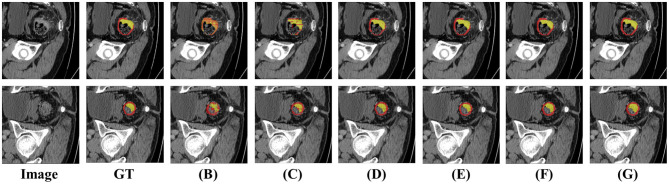
Visualization of performance comparisons using different combinations of model components. Each combination demonstrates the contribution of specific modules to the overall segmentation performance. Best viewed by zooming in. **(A–G)** Correspond to different configurations in the ablation study, as defined in Table 3.

To validate the effectiveness of each module in the proposed model, we conducted experiments on the CARE dataset, and the results are shown in Table 3 and Figure 7.

**Table 3:** Quantitative results of the ablation study on the CARE dataset.

**Configurations**	**Methods**						**CARE**	
	**LoRA**	**Adapter**	**PHFD**	**HFM**	**MHPM**	**ML**	**mDice(**%**)**	**mIoU(**%**)**
(A)	✕	✕	✕	✕	✕	✕	64.14	47.55
(B)	✓	✕	✕	✕	✕	✕	65.98	49.44
(C)	✓	✓	✕	✕	✕	✕	66.54	50.63
(D)	✓	✓	✓	✕	✕	✕	67.84	52.25
(E)	✓	✓	✓	✓	✕	✕	69.12	53.77
(F)	✓	✓	✓	✓	✓	✕	72.98	56.72
(G)	✓	✓	✓	✓	✓	✓	74.05	58.96

Different combinations of components are evaluated to assess their contributions to overall segmentation performance.

#### 4.6.1 Effects of LoRA and adapter

As shown in rows 1–3 of Table 3, the pre-trained SAM in zero-shot mode achieved an mIoU of 47.55% on the CARE dataset, further confirming the significant differences between natural images and medical images. After incorporating LoRA, the mIoU increased by 1.89%, and with the additional integration of the Adapter, the mIoU improved by a further 1.19%. The experimental results demonstrate that by introducing these two efficient fine-tuning mechanisms, the gap between natural and medical images can be effectively reduced, enabling the extraction of more robust feature information.

#### 4.6.2 Effects of key modules

As shown in rows 3–6 of Table 3, we validated the effectiveness of the key modules by incorporating them into the backbone network. By constructing the PHFD, the model can aggregate multi-level features and more comprehensively represent the overall structure of medical images, resulting in a 1.62% improvement in mIoU on the CARE dataset. After introducing HFM, the model effectively prevents excessive reliance on specific channels, thereby enhancing its generalization. The inclusion of HFM led to an additional 1.52% increase in mIoU. Finally, the MHPM further captures multi-scale information, which is particularly effective for segmenting lesions of different sizes, contributing to an additional 2.95% improvement in mIoU. Compared to SAM with LoRA and Adapter, incorporating all key modules resulted in a total improvement of 6.09% in mIoU on the CARE dataset.

#### 4.6.3 Effects of different losses

We validated the adequacy of model training by adjusting both the placement and the number of loss functions. As shown in Table 3, row 6 represents the model with the loss function applied only at the final output, while row 7 shows the loss function applied at all stages of the decoder. It can be observed that single-point supervision is insufficient for fully training the model. With the introduction of additional supervision, the model achieves better segmentation results.

#### 4.6.4 Effects of different combinations and *k* groups in HFM

**Figure 8:**
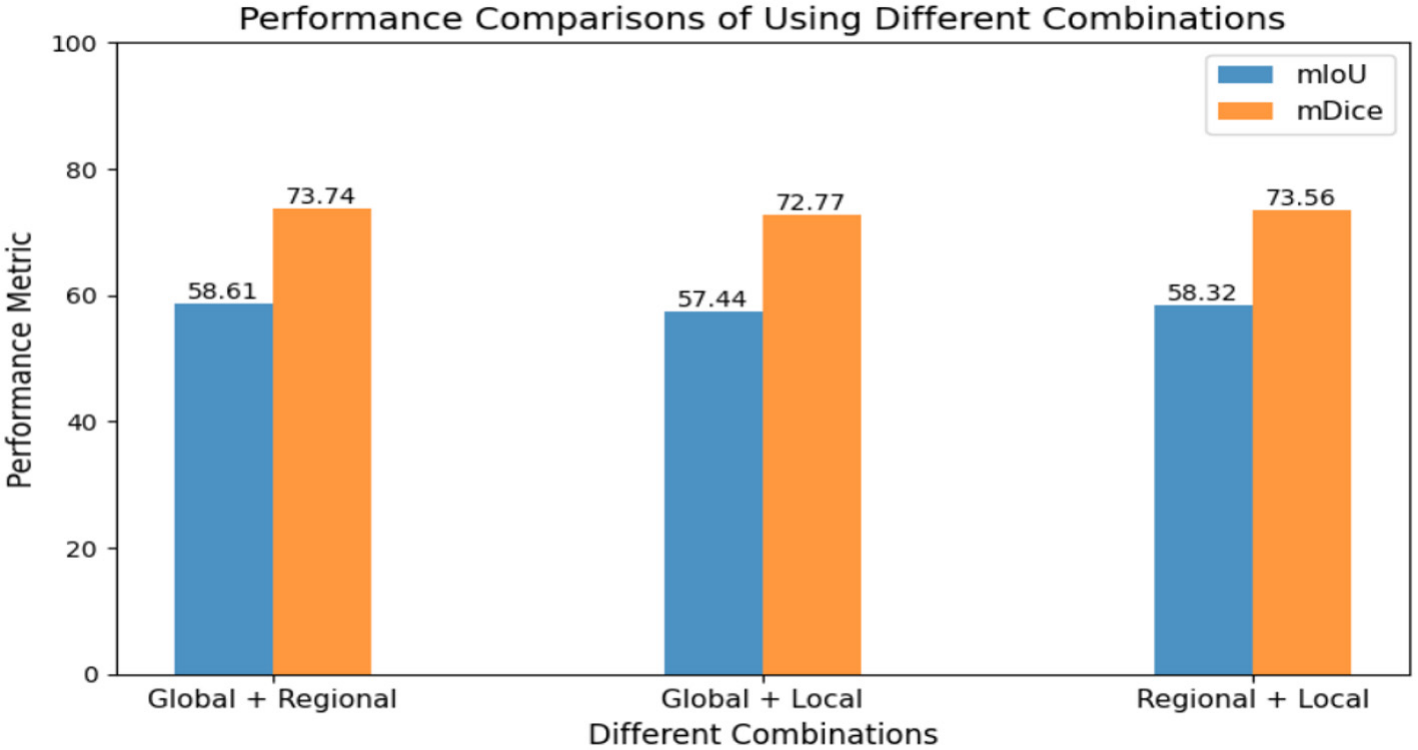
Performance comparisons of using different combinations.

In designing the HFM module to capture rich semantic information, we divided the input features into three levels: global, regional, and local, thereby enhancing the model's feature representation capability. Figure 8 shows the performance results with different combinations. It can be observed that when the regional branch is not introduced, the model achieves only 57.44% mIoU. This also indicates that including the regional branch prevents the model from relying on specific channels, thereby improving its generalization.

**Figure 9:**
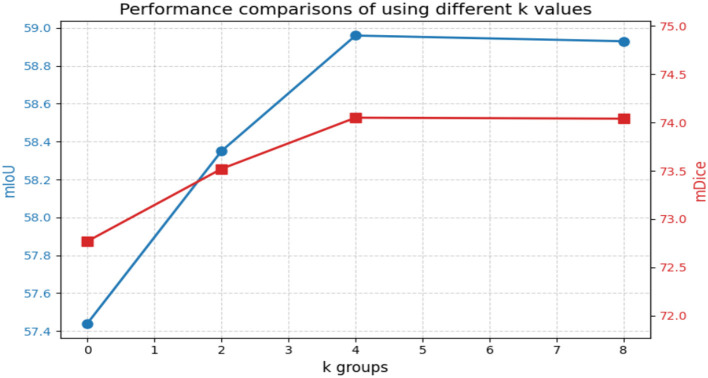
Performance comparisons of using different *k* values.

Figure 9 illustrates the impact of dividing the regional features into *k* groups on performance. The introduction of the regional branch enables the model to capture finer-grained information, thereby improving performance. In our work, we divided the regional features into four groups to strike a balance between accuracy and complexity. Specifically, we tested *k* values of 0, 2, 4, and 8. As shown in Figure 9, *k* = 0 (no regional branch) yields the lowest mIoU of 57.44%, confirming the importance of regional feature extraction. Increasing *k* to 2 improves mIoU to 58.35%, while *k* = 4 achieves the best performance at 58.96%. Further increasing *k* to 8 shows no additional improvement (58.93%), suggesting that excessive grouping may introduce redundancy. Therefore, *k* = 4 provides an optimal trade-off between feature granularity and computational efficiency.

#### 4.6.5 Effects of input resolution

To investigate the impact of input resolution on segmentation performance, we conducted experiments with different input sizes on the CARE dataset. As shown in Table 4, higher input resolutions lead to notable performance improvements. Since the CARE dataset contains rich, fine-grained details in lesion boundaries, increasing the resolution significantly enhances segmentation accuracy. Specifically, the 512 × 512 resolution improves mDice by 1.07% and mIoU by 0.85% compared to 224 × 224, while the 1,024 × 1,024 resolution achieves gains of 2.30% in mDice and 1.83% in mIoU. However, considering a fair comparison with previous methods (Oktay et al., 2018; Jha et al., 2019; Ibtehaz and Rahman, 2020) and the substantial computational overhead at higher resolutions, we maintain the same experimental settings (224 × 224) as prior works in our main experiments.

**Table 4:** Performance comparison of different input resolutions on the CARE dataset.

**Resolution**	**mDice(%)**	**mIoU(%)**	**GPU Memory (GB)**
224 × 224	74.05	58.96	14.8
512 × 512	75.12	59.81	32.6
1,024 × 1,024	76.35	60.79	58.4

### 4.7 Computational cost

To highlight the computational advantages, we compared the number of trainable parameters across several typical methods. As shown in Table 5, the SAM model, without using any prompt information (e.g., points or boxes), has 90.21 million trainable parameters. U-SAM, which extends SAM's backbone by incorporating U-shaped adapters into both the encoder and decoder, has 103.36 million trainable parameters. In contrast, our approach uses SAM as the backbone and introduces MHPM and PHFD to generate highly accurate, detailed segmentation masks for colorectal cancer regions. Compared to U-SAM, our model adds only a small number of trainable parameters while achieving better segmentation performance.

**Table 5:** Comparison of the number of trainable parameters across several typical methods on the CARE dataset.

**Methods**	**mDice(*%*)**	**mIoU(*%*)**	**TParam(M)**
UCTransNet	67.10	50.59	66.24
TransUnet-B	65.45	48.87	93.23
SwinUnet-B	67.97	51.67	149.11
TransUnet-L	68.17	51.86	315.08
SwinUnet-L	67.12	50.77	335.26
SAM-B	65.98	49.44	90.21
U-SAM	69.28	53.11	103.36
HHF-SAM	74.05	58.96	113.11

## 5 Conclusion

In this study, we propose a novel feature-learning framework for rectal cancer segmentation, which we name HHF-SAM. Specifically, we use the pre-trained SAM as the backbone of the proposed model. To address the gap between natural and medical images, we freeze the parameters of the original SAM encoder and introduce two efficient fine-tuning mechanisms. Subsequently, we incorporate the MHPM module, which employs a multi-scale feature-extraction mechanism to more effectively capture critical information in colonoscopy images. Finally, we propose a Progressive Hierarchical Fusion Decoder (PHFD) with a pyramid structure, which, combined with the Hierarchical Fusion Module (HFM), enables efficient segmentation predictions. In the future, we will explore further optimizations and integrate more advanced strategies to enhance the model's performance.

## Data Availability

The original contributions presented in the study are included in the article/supplementary material, further inquiries can be directed to the corresponding authors.
